# In Vitro Effect of Local Anesthetics on *Candida albicans* Germ Tube Formation

**DOI:** 10.1155/S1064744994000074

**Published:** 1994

**Authors:** Acácio Rodrigues, Cidália Pina Vaz, A. Freitas Fonseca, J. Martinez de Oliveira, Henrique Barros

**Affiliations:** 1 Department of Microbiology Porto Faculty of Medicine University of Porto Porto Portugal; 2 Department of Gynecology Porto Faculty of Medicine University of Porto Porto Portugal; 3 Department of Hygiene and Epidemiology Porto Faculty of Medicine University of Porto Porto Portugal; 4 Department of Microbiology Porto Faculty of Medicine Alameda Prof. Herani Monteiro Porto 4200 Portugal

## Abstract

*Objective:* This study was planned to clarify the in vitro effect of lidocaine and bupivacaine on germ tube formation by *Candida albicans* isolates from cases of clinical vaginal candidiasis.

*Methods:* Fourteen *C. albicans* strains (clinical vaginal isolates) were grown on Sabouraud agar for 24 h at 37℃ and tested as follows: 100 μl of a yeast suspension [10^5^ colony forming units (CFU)/ml of phosphate buffered saline (PBS)] was added to 500 μl of fresh human serum with lidocaine or bupivacaine (pure salts) in serial concentrations. The test was run in duplicate. Controls were prepared for each strain. After 4 h of incubation at 37℃, samples were taken from each vial and 200 yeasts were counted in a counting chamber. The pH of each suspension was measured.

*Results:* The results are given as the mean of the 2 readings and are expressed as the percentage of blastoconidia with germ tubes/total blastoconidia.

*Conclusions:* Our experiments show that both lidocaine and bupivacaine have a dose-dependent inhibitory effect, pH-independent, on germ tube formation by *C. albicans* and that both drugs seem to be promising in the treatment of genital candidiasis due to the combination of anesthetic and antifungal properties.


Infectious Diseases in Obstetrics and Gynecology 1:193–197 (1994)
© 1994 Wiley-Liss, Inc.

### In Vitro Effect of Local Anesthetics on *Candida albicans* Germ Tube Formation

**Acácio Rodrigues, Cidália Pina Vaz, A. Freitas Fonseca, J. Martinez de Oliveira, and Henrique Barros**

*Departments of Microbiology (A.R., C.P.V., A.F.F.), Gynecology (J.M.d.O.), and Hygiene and Epidemiology (H.B.), Porto Faculty of Medicine, University of Porto, Porto, Portugal*

Abstract

***Objective:*** **This study was planned to clarify the in vitro effect of lidocaine and bupivacaine on germ tube formation by *Candida albicans* isolates from cases of clinical vaginal candidiasis.**

***Methods:*** **Fourteen *C. albicans* strains (clinical vaginal isolates) were grown on Sabouraud agar for 24 h at 37°C and tested as follows: 100 μl of a yeast suspension [$10^5$ colony forming units (CFU)/ml of phosphate buffered saline (PBS)] was added to 500 μl of fresh human serum with lidocaine or bupivacaine (pure salts) in serial concentrations. The test was run in duplicate. Controls were prepared for each strain. After 4 h of incubation at 37°C, samples were taken from each vial and 200 yeasts were counted in a counting chamber. The pH of each suspension was measured.**

***Results:*** **The results are given as the mean of the 2 readings and are expressed as the percentage of blastoconidia with germ tubes/total blastoconidia.**

***Conclusions:*** **Our experiments show that both lidocaine and bupivacaine have a dose-dependent inhibitory effect, pH-independent, on germ tube formation by *C. albicans* and that both drugs seem to be promising in the treatment of genital candidiasis due to the combination of anesthetic and antifungal properties.** © 1994 Wiley-Liss, Inc.

Key words

Local anesthetics, germ tube, antimicrobial activity, candidiasis

In 1909 Jonnesco[1] first described the antibacterial effect of local anesthetic drugs and in 1955 Murphy et al.[2] reported the toxicity of tetracaine in relation to *Pseudomonas*. Kleinfeld and Ellis[3,4] could demonstrate a toxic effect of tetracaine, benoxinate, and cocaine to *Candida albicans*. Erlich[5] showed that, while tetracaine at a 0.5% concentration inhibited *C. albicans* as well as *Staphylococcus aureus*, greater concentrations of lidocaine were required to obtain a similar effect. In 1970 Schmidt and Rosenkranz[6] demonstrated the antimicrobial activity of lidocaine and procaine against several bacteria and yeasts. These authors did not study any genital specimens. Of 10 *Cryptococcus neoformans* and *C. albicans* studied (isolates from nongenital sources), all 5 *Cryptococcus* were inhibited by lidocaine and procaine in concentrations in excess of 1.0%. However, none of the 5 *Candida* strains were inhibited by 2.0% lidocaine or procaine. Meanwhile, all 10 isolates were inhibited by 0.5–1.0 μg/ml of amphotericin B.[6] Therefore, the results of Schmidt and Rosenkranz[6] regarding *C. albicans* are in disagreement with those of previous authors.

It is admitted that filamentation of *Candida* blastoconidia is a phenotypical change related to pathogenesis.[7,8] The presence of hyphae on the bedside "wet smear" examination is supposed to indicate invasiveness and an abnormal stage not accepted as colonization.[9] Some years ago, Taschdjian et al.[10] used germ tube formation as a test for identification

Address correspondence/reprint requests to Dr. Acácio Rodrigues, Department of Microbiology, Porto Faculty of Medicine, Alameda Prof. Hernani Monteiro, 4200 Porto, Portugal.

Received August 12, 1993
Accepted January 5, 1994

Basic Science Article

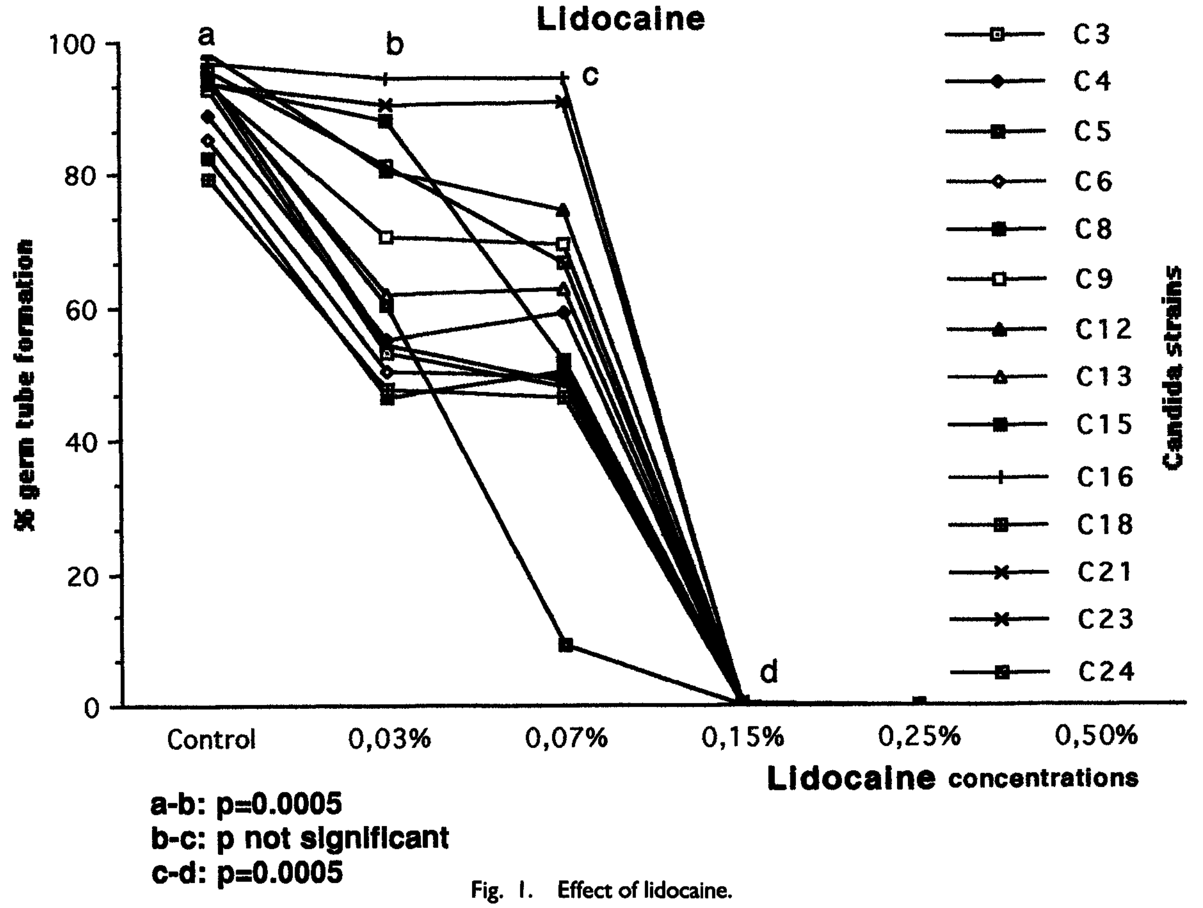

Fig. 1. Effect of lidocaine.

of *C. albicans*. In addition to antifungal properties, local anesthetics could help in relieving the very stressful symptoms commonly present in genital candidiasis. The present work was performed to clarify the in vitro effect of 2 local anesthetics, lidocaine, a common and well-known agent available for topical use, and bupivacaine, a more recent and potent drug, on the germ tube formation by *C. albicans*.

### MATERIALS AND METHODS

All 14 *C. albicans* strains used in this study were clinical isolates from microbiologically diagnosed cases of vaginal candidiasis, some of them from recalcitrant cases or cases resistant in vivo to several antifungal treatments. They were grown on Sabouraud agar (Difco, Detroit, MI) for 24 h at 37°C and tested as follows: 100 μl of a yeast suspension [$10^5$ colony forming units (CFU)/ml of phosphate buffered saline (PBS)] was added to 500 μl of fresh human serum (pool) with lidocaine or bupivacaine in serial concentrations. Solutions of lidocaine and bupivacaine hydrochloride (pure salts, free of preservatives, purchased from Sigma, St. Louis, MO) were prepared in sterile PBS immediately before experiments in order to guarantee good stability. For each concentration of both local anesthetics, the test was run in duplicate. Duplicate controls, with plain serum, were prepared with each strain. After 4 h of incubation at 37°C, the samples were taken from each vial and 200 yeasts were evaluated and counted using a Burke's chamber under a magnification of ×400 on a blind reading. The pH value of each suspension was measured. For the statistical analysis of data, we used the Wilcoxon signed rank tests.[11]

### RESULTS

Results are given as the mean of the 2 readings, for each strain and concentration, and are expressed as the percentage of blastoconidia with germ tubes/

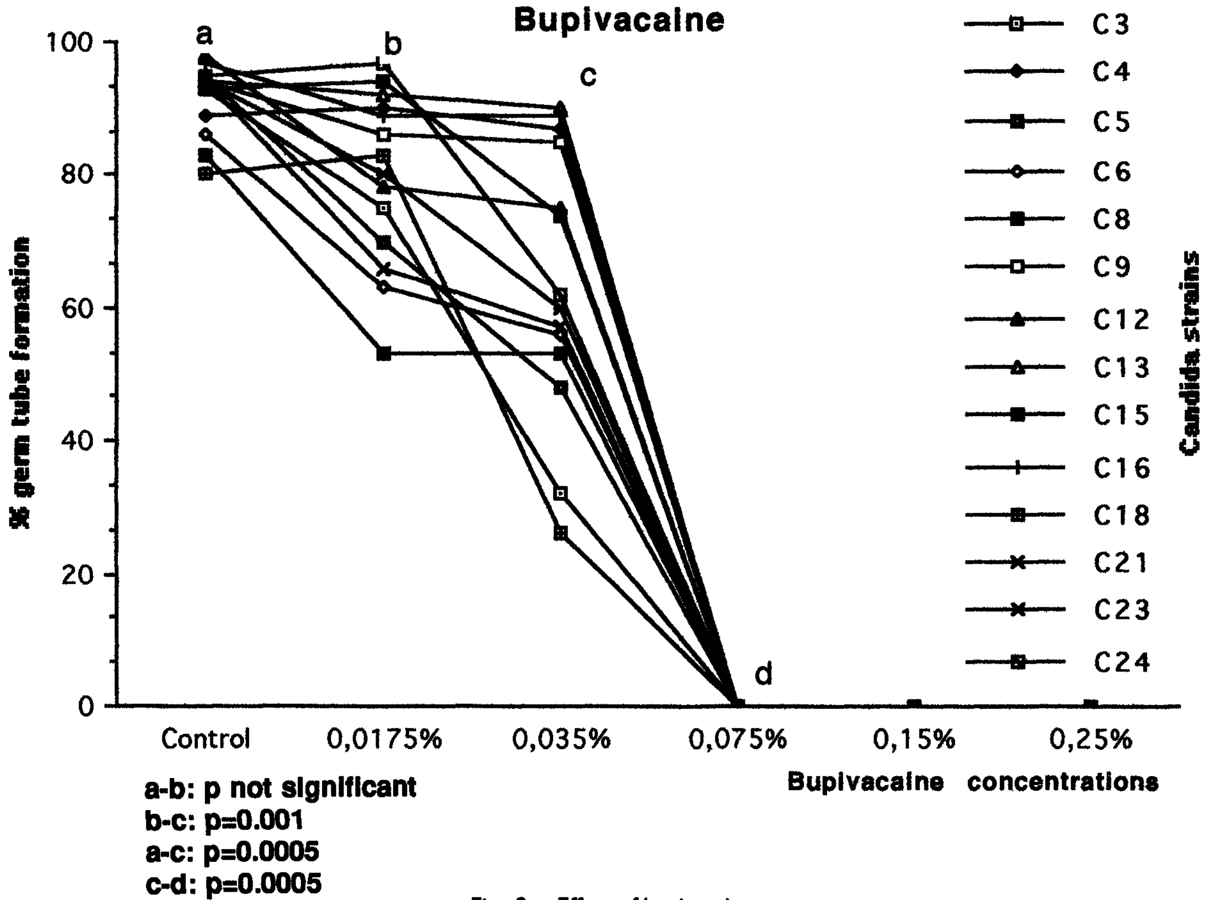

Fig. 2. Effect of bupivacaine.

total blastoconidia. No significant pH variation was found between controls and local anesthetic solutions.

The effect of lidocaine is shown in Figure 1 and the effect of bupivacaine in Figure 2. The degree of germ tube inhibition of each local anesthetic at different concentrations is listed in Table 1.

### DISCUSSION

The mechanisms involved in *Candida* pathogenesis are not yet well defined, even in *C. albicans*, which is commonly accepted as the most pathogenic species.[12] In contrast to many bacteria, no single virulence factor can be identified as responsible for fungal infectivity. Yeast pathogenicity results from several different factors, the resultant being the overwhelming of the host defenses by the yeasts. Although not a crucial step, germ tube formation is believed to play an important pathogenic role in the initial phase of tissue invasion. In some studies, germ tubes were proved to be associated with better adhesion to epithelial cells in vitro,[13,14] and it has been repeatedly shown in vitro that *C. albicans* germ tubes grow from macrophages[15,16] as they do from neutrophils (PMNs)[17] after phagocytosis, in this way preventing the intracellular killing of the yeast. These and several other experimental facts helped to create the concept of a differential behavior of *C. albicans* germ tubes and pseudohyphae in relation to blastoconidia. The reason why *C. albicans* is a common and persistent pathogen in the vaginas of a significant number of women[18–20] still remains undetermined.

In our study, we decided to use serum for the germ tube test due to the more impressive germ tube production in this substrate when compared to others. In a previous study (unpublished data), we evaluated the different sensitivities for germ tube formation of several media, namely Eagle's medium, RPMI medium, Sabouraud's dextrose broth, egg albumin, fetal calf serum, and human serum,

**TABLE 1. Percent of germ tube formation inhibition at serial concentrations of local anesthetic**

| Concentration (%) | Lidocaine (%) | Bupivacaine (%) |
|---|---|---|
| 0.0175 | — | 12.1 ± 12.0 |
| 0.035 | 24.0 ± 13.8 | 27.9 ± 18.4 |
| 0.075 | 33.0 ± 20.0 | 91.8 ± 5.3 |
| 0.15 | 91.6 ± 5.5 | 100 |
| 0.25 | 100 | 100 |
| 0.50 | 100 | 100 |

the latter being the most sensitive. That human serum (a pool, to avoid possible anticandidal factors present in a particular serum that might affect germ tubes) is a good substrate for germ tube production is also confirmed in this work, being the mean value of germ tube formation in our controls (plain human serum), 91.8 ± 5.3%.

Our results show that both lidocaine and bupivacaine have a potent dose-dependent inhibitory effect on germ tube formation by *C. albicans*. This effect is pH-independent, as there is no significant pH variation when compared to controls. From the values we found, bupivacaine seems to be more potent on that action than lidocaine. In fact, when the results are compared for the 0.07% concentration (in which there is a significant effect of both drugs), bupivacaine seems to be 3 times more potent than lidocaine (91.8 ± 5.3% vs. 33.0 ± 20.0% of inhibition; $P = 7.3 \times 10^{-6}$) in achieving that effect. In very dilute concentrations such as 0.15% (compared to the ones used in clinical practice for several purposes), we found 100% inhibition of germ tube formation with bupivacaine and almost the same figure with lidocaine (91.6 ± 5.5%). When the 0.25% concentration is considered, both lidocaine and bupivacaine produced an inhibition of 100%. Similarly, low concentration values may be easily attainable in the vaginal milieu, in vivo, probably with similar effects. The mechanism by which local anesthetics exert their antimicrobial effects is still unclear to us. A plausible hypothesis is the impairment of protein synthesis necessary to germ tube formation, namely, the cell wall or the cytoplasmic membrane. Biochemical studies demonstrated that protein synthesis was more sensitive than RNA or DNA production to the inhibitory effect of lidocaine, although no distinct selective inhibition of macromolecular synthesis was found.[6] Further testing is being carried out to clarify this effect. To our knowledge, this is the first report of the effect of local anesthetics, namely lidocaine and bupivacaine, on germ tube formation by *C. albicans*. From a theoretical point of view, these drugs seem to be promising in the treatment of genital candidiasis due to the combination of anesthetic and antifungal properties.

### REFERENCES

1. Jonnesco T: Remarks on general spinal analgesia. Br Med J 2:1396–1401, 1909.
2. Murphy JT, Allen HF, Mangiaracine AB: Preparation, sterilization, and preservation of ophthalmic solutions: Experimental studies and a practical method. Arch Ophthalmol 53:63–78, 1955.
3. Kleinfeld J, Ellis PP: Effects of topical anesthetics on growth of microorganisms. Arch Ophthalmol 76:712–725, 1966.
4. Kleinfeld J, Ellis PP: Inhibition of microorganisms by topical anesthetics. Appl Microbiol 15:1296–1298, 1967.
5. Erlich H: Bacteriologic studies and effects of anesthetic solutions on bronchial secretions during bronchoscopy. Am Rev Respir Dis 84:414–421, 1961.
6. Schmidt RM, Rosenkranz HS: Antimicrobial activity of local anesthetics: Lidocaine and procaine. J Infect Dis 121:587–607, 1970.
7. Sobel JD: Epidemiology and pathogenesis of recurrent vulvovaginal candidiasis. Am J Obstet Gynecol 152:924–935, 1985.
8. Odds FC: Morphogenesis in *Candida*, with special reference to *C. albicans*. In Odds FC (ed): *Candida* and Candidosis. Baillière Tindall, London, pp 42–59, 1988.
9. Eschenbach DA. Clin Obst Gynecol 26:186–201, 1983.
10. Taschdjian CL, Burchall JJ, Kozinn PJ: Rapid identification of *Candida albicans* by filamentation on serum and serum substitutes. Am J Dis Child 99:212–215, 1960.
11. Hill B: The Wilcoxon signed rank test. In Hill B (ed): Principles of Medical Statistics, 12th Ed. London: Edward Arnold, pp 126–127, 1991.
12. Hurley R: Pathogenicity of the genus *Candida*. In Winner HI, Hurley R (eds): Symposium on *Candida* Infections. Edinburgh: Churchill Livingstone, pp 13–25, 1966.
13. Kimura LH, Pearsall NM: Relationship between germination of *Candida albicans* and increased adherence to human buccal epithelial cells. Infect Immun 28:464–468, 1980.
14. Rotrosen D, Edwards JE, Gibson TR, et al.: Adherence of *Candida* to cultured vascular endothelial cells: Mechanisms of attachment and endothelial penetration. J Infect Dis 152:1264–1274, 1985.
15. Peterson EM, Calderone RA: Growth inhibition of *Candida albicans* by rabbit alveolar macrophages. Infect Immun 15:911–915, 1977.

16. Evron R: In vitro phagocytosis of *Candida albicans* by peritoneal mouse macrophages. Infect Immun 28:963–971, 1980.
17. Arai T, Mikami Y, Yokohama K: Phagocytosis of *Candida albicans* by rabbit alveolar macrophages and guinea pig neutrophils. Sabouraudia 15:171–177, 1977.
18. Sobel JD: Epidemiology and pathogenesis of recurrent vulvovaginal candidiasis. Am J Obstet Gynecol 152:924–935, 1985.
19. Sobel JD: Recurrent vulvovaginal candidiasis: What we know and what we don't. Ann Intern Med 101:390–392, 1984.
20. Martinez de Oliveira J, Silva Cruz A, Fonseca AF, Pina Vaz C, Rodrigues A: Prevalence of *Candida albicans* in the vaginal fluid of Portuguese women. J Reprod Med 38:41–42, 1993.
